# Single-shot real-time femtosecond imaging of temporal focusing

**DOI:** 10.1038/s41377-018-0044-7

**Published:** 2018-08-08

**Authors:** Jinyang Liang, Liren Zhu, Lihong V. Wang

**Affiliations:** 1 Caltech Optical Imaging Laboratory, Andrew and Peggy Cherng Department of Medical Engineering, Department of Electrical Engineering, California Institute of Technology, 1200 East California Boulevard, Mail Code 138-78, Pasadena, CA 91125 USA; 2 Present Address: Centre Énergie Matériaux Télécommunications, Institut National de la Recherche Scientifique, 1650 Boulevard Lionel-Boulet, Varennes, QC J3X1S2 Canada

## Abstract

While the concept of focusing usually applies to the spatial domain, it is equally applicable to the time domain. Real-time imaging of temporal focusing of single ultrashort laser pulses is of great significance in exploring the physics of the space–time duality and finding diverse applications. The drastic changes in the width and intensity of an ultrashort laser pulse during temporal focusing impose a requirement for femtosecond-level exposure to capture the instantaneous light patterns generated in this exquisite phenomenon. Thus far, established ultrafast imaging techniques either struggle to reach the desired exposure time or require repeatable measurements. We have developed single-shot 10-trillion-frame-per-second compressed ultrafast photography (T-CUP), which passively captures dynamic events with 100-fs frame intervals in a single camera exposure. The synergy between compressed sensing and the Radon transformation empowers T-CUP to significantly reduce the number of projections needed for reconstructing a high-quality three-dimensional spatiotemporal datacube. As the only currently available real-time, passive imaging modality with a femtosecond exposure time, T-CUP was used to record the first-ever movie of non-repeatable temporal focusing of a single ultrashort laser pulse in a dynamic scattering medium. T-CUP’s unprecedented ability to clearly reveal the complex evolution in the shape, intensity, and width of a temporally focused pulse in a single measurement paves the way for single-shot characterization of ultrashort pulses, experimental investigation of nonlinear light-matter interactions, and real-time wavefront engineering for deep-tissue light focusing.

## Ultrafast photography: World's fastest camera glimpses laser pulses at ten trillion frames per second

Improvements in a photography technique allows seeing instantaneous light patterns in real time. While standard optical instruments focus on a spatial point, temporal microscopes confine photons along a narrow plane that can penetrate samples and excite multiple components simultaneously. Lihong Wang from the California Institute of Technology in Pasadena, U.S.A. and colleagues have now developed the world’s fastest camera that can capture the temporal focus dynamics by taking trillions of images per second in a single exposure. The team’s setup records a sample’s dynamic intensity patterns, then splits them into two optical pathways retaining spatial and temporal information. Aided by compressed imaging and a fast streak camera, this division helps minimize the number of measurements and enables image reconstruction algorithms to achieve frame rates two orders of magnitude better than current receive-only ultrafast cameras.

## Introduction

The space–time duality in optics originates from the mathematical equivalence between paraxial diffraction and dispersive propagation^1^. Remarkably, this duality enables one to translate spatial-domain optical techniques to the temporal domain, which has fostered the development of powerful temporal imaging approaches, such as temporal microscopy, to characterize optical signals^2,3^. Among the many temporal imaging phenomena, temporal focusing, as a time-domain counterpart of spatial focusing, describes an exquisite optical phenomenon—temporal compression of the duration of a chirped laser pulse to the shortest time possible at a designated location^4–6^. Temporal focusing has been leveraged in the temporal 4*f* processor^7^ and the dispersive Fourier transformer^8^ for analyzing optical waveforms with unprecedented bandwidths. Akin to spatial focusing—confining photons laterally—temporal focusing enables photon confinement in the longitudinal direction. This salient feature has powered depth-sectioning wide-field nonlinear microscopy for neuroimaging^9^. Recently, temporal focusing has been achieved through static scattering media^10^, which has sparked interest in deep biomedical imaging. In addition, the strong intensity localization has made it an attractive tool for material processing^11^, which has led to extensive studies of elusive physics mechanisms of the strong-field interaction with matter^12^. Considering the stochastic (e.g., time reversal of dynamic speckle patterns to produce temporal focusing in live biological tissue^13^) and non-repeatable (e.g., micromachining using temporal focusing in glass^14^) nature of these transient phenomena, visualizing temporal focusing in real time (i.e., at its actual time of occurrence) becomes a prerequisite for investigating and further utilizing them. In addition, since the width and intensity of the laser pulse experiences drastic changes during temporal focusing, a femtosecond-level exposure time is required to clearly resolve the evolving instantaneous spatiotemporal details of this phenomenon. Moreover, the nanometer-to-micrometer spatial scales of these transient events demand ultrafast imaging for blur-free observation [e.g., for imaging a light-speed event, an imaging speed of 1 trillion frames-per-second (Tfps) is required for a spatial resolution of 300 µm]^15^. Finally, since these events are often self-luminescent, a passive (i.e., receive-only) detector is highly desired for direct recording.

Existing ultrafast imaging techniques, however, are incapable of providing real-time, femtosecond, passive imaging capability. The current mainstream technique used in ultrafast imaging is based on pump–probe measurements^16,17^. Although having achieved femtosecond temporal resolution and passive detection, these multiple-shot imaging techniques depend on precise repetition of the targeted ultrafast event during temporal or spatial scanning. Hence, in cases where temporal focusing must be recorded in a single measurement, these imaging techniques are inapplicable.

Recently, a number of single-shot ultrafast imaging techniques^18–22^ have been developed. Among them, active-illumination-based approaches have achieved frame rates at the Tfps level^20,21^. However, such approaches are incapable of imaging luminescent transient events, so they are precluded from direct imaging of evolving light patterns in temporal focusing. The requirement of active illumination was recently eliminated by a new single-shot ultrafast imaging modality, termed compressed ultrafast photography (CUP)^23–25^. Synergizing compressed sensing and streak imaging, CUP works by first compressively recording a three dimensional (3D, i.e., *x,y,t*) scene into a two-dimensional (2D) snapshot and then computationally recovering it by solving an optimization problem. The resultant CUP system can passively receive photons scattered or emitted from dynamic scenes at frame rates of up to 100 billion fps. CUP has been applied to a number of applications, including fluorescence lifetime mapping^23^, real-time imaging of a propagating photonic Mach cone^24^, and time-of-flight volumetric imaging^25^. However, in these previous studies, the frame interval (defined as the reciprocal of the frame rate) was 10 ps, which has hindered the use of CUP for imaging spatiotemporal details of temporal focusing in the femtosecond regime.

## Results

### Principle and system of T-CUP

**Fig. 1:**
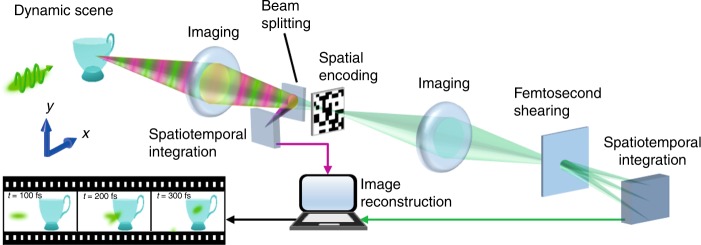
Principle of operation for T-CUP. The beam paths for time-unsheared and time-sheared views are illustrated using magenta and green colors, respectively

To enable real-time, ultrafast, passive imaging of temporal focusing, here, we have developed single-shot trillion-frame-per-second compressed ultrafast photography (T-CUP), which can image non-repeatable transient events at a frame rate of up to 10 Tfps in a receive-only fashion. The operation of T-CUP consists of data acquisition and image reconstruction (Fig. 1). For the data acquisition, the intensity distribution of a 3D spatiotemporal scene, *I*[*m,n,k*], is first imaged with a beam splitter to form two images. The first image is directly recorded by a 2D imaging sensor via spatiotemporal integration (defined as spatial integration over each pixel and temporal integration over the entire exposure time). This process, which forms a time-unsheared view with an optical energy distribution of *E*_u_[*m*,*n*], can be expressed by

1 $$E_{\rm{u}}\left[ {m,n} \right] = \eta \mathop {\sum }\limits_k \left( {h_{\rm{u}} \ast I} \right)\left[ {m,n,k} \right]$$

where *η* is a constant, *h*_u_ represents spatial low-pass filtering imposed by optics in the time-unsheared view, and * denotes the discrete 2D spatial convolution operation. Equation 1 can be regarded as a single-angle Radon transformation operated on *I*[*m, n, k*] (detailed in Supplementary Note 1).

The second image is spatially encoded by a pseudo-random binary pattern. Then the spatially encoded scene is relayed to a femtosecond shearing unit, where temporal frames are sheared on one spatial axis. Finally, the spatially encoded, temporally sheared frames are recorded by another 2D imaging sensor via spatiotemporal integration to form a time-sheared view with an optical energy distribution of *E*_s_[*m*,*n*]. This process can be described by

2 $$E_{\rm{s}}\left[ {m,n} \right] = \eta \mathop {\sum }\limits_k \left( {h_{\rm{s}} \ast I_{\rm{C}}} \right)\left[ {f_{\rm{D}},g_{\rm{D}} + k,k} \right]$$

where *h*_s_ represents spatial low-pass filtering in the time-sheared view. *I*_C_[*f*_D_,*g*_D_*+k*,*k*] is the spatially encoded scene. *f*_D_ and *g*_D_ are the discrete coordinates transformed from *m* and *n*, according to the distortion in the time-sheared view^24^. Equation 2 can be regarded as the Radon transformation of the spatiotemporal datacube from an oblique angle determined by the shearing speed of the streak camera and pixel size of the sensor (detailed in Supplementary Note 1).

Combining the two views, the data acquisition of T-CUP can be expressed by a linear equation,

3 $$\left[ {E_{\rm{u}},\alpha E_{\rm{s}}} \right]^T = \left[ {{\boldsymbol{O}}_{\rm{u}},\alpha {\boldsymbol{O}}_{\rm{s}}} \right]^TI$$

where *α* is a scalar factor introduced to balance the energy ratio between the two views during measurement, and ***O***_u_ and ***O***_s_ are the measurement operators for the two views (see Materials and methods and Supplementary Fig. S1). Thus T-CUP records a 3D dynamic scene into two 2D projections in a single exposure.

Image reconstruction of the scene can be done by solving the minimization problem of $\min _I\left\{ {\frac{1}{2}\Vert \left[ {E_{\rm{u}},\alpha E_{\rm{s}}} \right]^T - \left[ {{\boldsymbol{O}}_{\rm{u}},\alpha {\boldsymbol{O}}_{\rm{s}}} \right]^TI \Vert_2^2 + \rho {\it{\Phi }}\left( I \right)} \right\}$, where $\left\| \cdot \right\|$ denotes the *l*^2^ norm, Ф(*I*) is a regularization function that promotes sparsity in the dynamic scene, and *ρ* is the regularization parameter (detailed in Supplementary Notes 2). The solution to this minimization problem can be stably and accurately recovered, even with a highly compressed measurement^26^.

The integration of compressed sensing into the Radon transformation drastically reduces the required number of projections to two. The time-unsheared view, in which the projection is parallel to the time axis, losslessly retains spatial information while discarding all temporal information. The time-sheared view, on the other hand, preserves temporal information by projecting the spatiotemporal datacube from an oblique angle. As a result, these two views, as an optimal combination, enable one to record an optimum amount of information with the minimum number of measurements. However, a direct inversion of the Radon transform is not possible in this case due to the small number of projections and the fact that the linear system (Eq. 3) that needs to be inverted is under-determined. To solve this problem, compressed sensing is used. Leveraging the sparsity of the scene, as well as the random encoding in the time-sheared view as prior information, the compressed-sensing-based reconstruction algorithm uses the regularization-function-guided search to find a unique solution. Our simulation has demonstrated that this compressed-sensing-augmented two-view projection can retrieve a dynamic scene with a high reconstruction quality (Supplementary Fig. S2 and detailed in Supplementary Note 3).

**Fig. 2:**
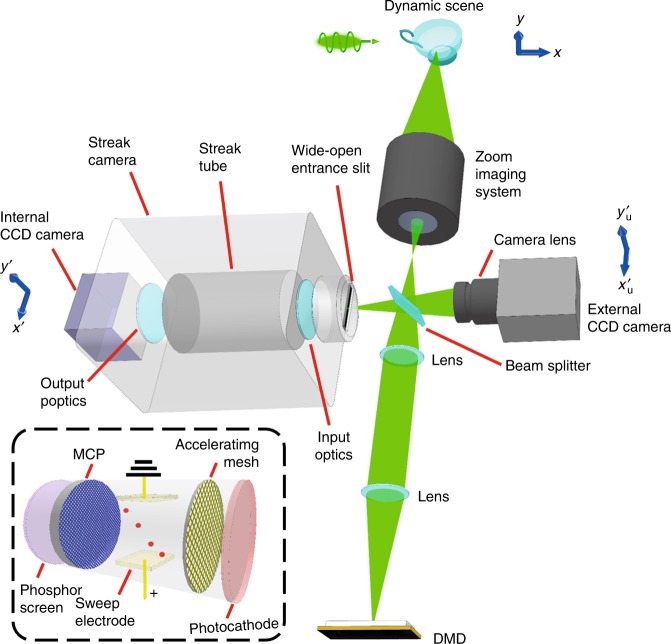
Schematic of the T-CUP system. Inset (black dashed box): detailed illustration of the streak tube. CCD charge-coupled device, DMD digital micromirror device, MCP micro-channel plate

In practice, T-CUP is embodied in an imaging system (Fig. 2 and detailed in Materials and methods) that uses several key devices to realize specific operations. Specifically, a charge-coupled device (CCD) camera performs spatiotemporal integration, a digital micromirror device (DMD) performs spatial encoding, and the time-varying voltage applied to the sweep electrodes in a femtosecond streak camera accomplishes femtosecond shearing. In addition, a compressed-sensing-based two-view reconstruction algorithm recovers the dynamic scene. The T-CUP system can capture a dynamic scene with spatial dimensions of 450 × 150 pixels and a sequence depth (i.e., number of frames per movie) of 350 frames in a single camera exposure. The frame rate of the reconstructed video is determined by *v*/*d*, where *v* is the temporal shearing velocity of the streak camera, and *d* is the pixel size of the internal CCD along the temporal shearing direction. By varying *v*, the frame rate can be widely adjusted from 0.5 to 10 Tfps. Thus, with single-shot data capture, a tunable ultrahigh frame rate, and an appreciable sequence depth, the T-CUP system is well suited for imaging single-event ultrafast transient phenomena occurring over a wide range of timescales (the characterization of the spatial and temporal resolutions of T-CUP is detailed in Supplementary Fig. S3 and Supplementary Note 4). The T-CUP temporal resolutions for 0.5, 1, 2.5, and 10 Tfps frame rates have been quantified to be 6.34, 4.53, 1.81, and 0.58 ps, respectively.

### Imaging temporal focusing of a single femtosecond laser pulse using the T-CUP system

**Fig. 3:**
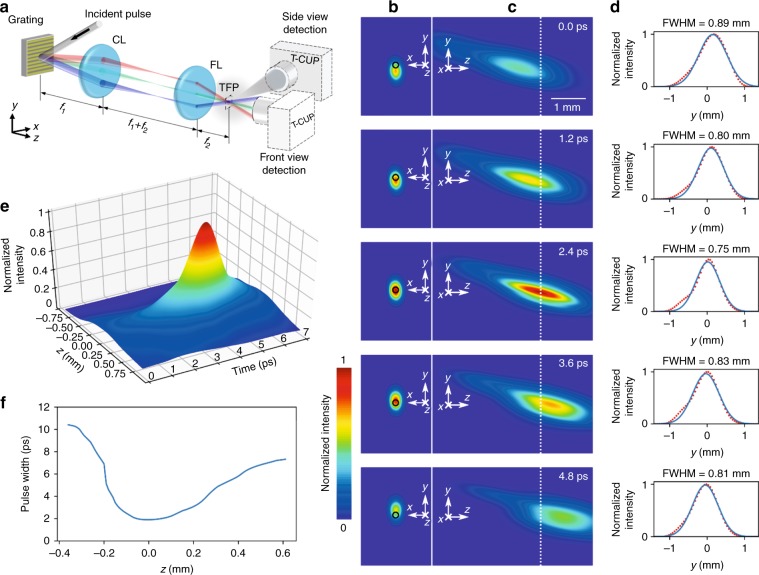
T-CUP for temporal focusing. **a** Experimental setup. CL collimating lens, FL focusing lens, TFP temporal focusing plane, *f*_1_ and *f*_2_ focal lengths. For the side view, a small amount of water vapor was used to scatter photons into the T-CUP system. **b** Representative frames from the front view (see Supplementary Movie S1 for the full evolution), showing the laser pulse sweeping along the *y* axis of the temporal focusing plane. The black circles denote the *z* axis. **c** Representative frames from the side view (see Supplementary Movies S1 and S2 for the full evolution), showing a single ultrashort laser beam propagating through the temporal focusing plane. The temporal focusing plane is marked by the dashed line. **d** Light intensity projected along the *z* axis, averaged and normalized individually, for each frame in **c**. The red dots are the data measured from the images (to avoid cluttering, only one data point is shown for every five data points measured and used for fitting), and the blue curves show the Gaussian fittings to the measured data. Full widths at half maxima (FWHMs) are computed for each fitted curve. **e** Surface plot of the normalized intensity along the primary optical axis as a function of *t* and *z* near the temporal focusing plane. **f** Measured pulse widths (FWHMs) as a function of position on the *z* axis near the temporal focusing plane. The location of the temporal focusing plane is set to be zero

A typical temporal focusing setup consists of a diffraction grating and a 4*f* imaging system (Fig. 3a). The incident laser pulse is first spatially dispersed by the grating and then collected by a collimation lens. Finally, a focusing lens recombines all the frequencies at the focal plane of the lens (Supplementary Fig. S4 and detailed in Supplementary Note 5). Temporal focusing has two major features: first, the shortest pulse width is at the focal plane of the focusing lens^4^; second, the angular dispersion of the grating creates a pulse front tilt so that the recombined pulse scans across the focal plane^5^. The pulse front tilt angle can be expressed by $\gamma = \tan ^{ - 1}\left( {\lambda _{\rm{c}}/Md_{\rm{g}}} \right)$ (refs. ^27,28^), where *M* is the overall magnification ratio, *λ*_c_ is the central wavelength of the ultrashort pulse, and *d*_g_ is the grating period. The femtosecond pulse that undergoes temporal focusing presents a complex spatiotemporal profile (Supplementary Fig. S4) that can be revealed only in the captured instantaneous light patterns. Even a picosecond-level exposure time would erase these spatiotemporal details via significant temporal blurring. This speed requirement excludes previous CUP systems^23–25^ from visualizing this ultrafast optical phenomenon. In contrast, T-CUP can achieve unprecedented real-time visualization with a single camera exposure.

We imaged the temporal focusing from both the front and the side (Fig. 3a) at 2.5 Tfps. A collimated femtosecond laser pulse (800 nm central wavelength, 50 fs pulse duration, 1 × 3 mm^2^ spatial beam size) was used to illuminate a 1200 line mm^−1^ grating. The 4*f* imaging system had a magnification ratio of *M*=1/4. In theory, the tilt angle for the pulse front at the temporal focusing plane was 75.4°.

For front-view detection, T-CUP captured the impingement of the tilted laser pulse front sweeping along the *y* axis of the temporal focusing plane (Fig. 3b and Supplementary Movie S1). The pulse swept a distance of ~0.75 mm over 10 ps, corresponding to a pulse front tilt of ~76°, which closely matches the theoretical prediction.

For side-view detection, weak water vapor was spread as a dynamic scattering medium. T-CUP revealed the full evolution of the pulse propagation across the temporal focusing plane (Fig. 3c, d, Supplementary Fig. S5, and Supplementary Movies S1 and S2): a tilted pulse propagates toward the right. As it approaches the temporal focusing plane, the pulse width continuously reduces, manifesting as an increasing intensity. At the temporal focusing plane, the focus of the pulse sweeps along the *y* axis at its peak intensity. The evolution after the temporal focusing plane mirrors the preceding process: the pulse width is elongated, and the intensity is continuously weakened. We then quantitatively analyzed the pulse compression effect of temporal focusing. Figure 3e shows the temporal profiles of the laser pulse on the *z* axis near the temporal focusing plane, demonstrating the sharp temporal focusing of the laser pulse. Figure 3f shows the pulse duration along the *z* axis near the temporal focusing plane. The full width at half maximum of the temporal profile is reduced from 10.4 ps to 1.9 ps—compressed by a factor of 5.5. It is notable that the measured pulse width is wider than the incident pulse, which is likely due to dispersion by optical elements and scattering, as well as to the temporal broadening caused by the finite temporal resolution of the T-CUP system.

T-CUP is currently the only technology capable of observing temporal focusing in real time. First, the entire process of the imaged temporal focusing event occurred in ~10 ps, which equals the previous state-of-the-art exposure time for a single frame^23^; hence, it could not be resolved previously. In contrast, T-CUP, using a frame interval of 0.4 ps, clearly resolved the intensity fluctuation, width compression, and structural change of the temporal focusing process. Second, the dynamic scattering induced by the water vapor makes the scattered temporal focusing pulse non-repeatable. In different measurements, the reconstructed results show a difference in spatial shape, compression ratio, and intensity fluctuation. To demonstrate the non-repeatability, another dataset for the sideways detection of temporal focusing is shown in Supplementary Fig. S6.

Although the ultrashort laser pulse was dispersed and converged in space by the 4*f* imaging system, it is worth noting that the effect of spatial focusing is limited. As the pulse approached the temporal focusing plane, the beam size fluctuated with a normalized standard deviation of 5.6% over a duration of 4.8 ps (Fig. 3d), while the peak on-axis intensity of the pulse increased approximately five-fold (Fig. 3e). Thus the intensity increase is caused dominantly by the temporal focusing.

### Imaging light-speed phenomena in real time in both the visible and near-infrared spectral ranges

**Fig. 4:**
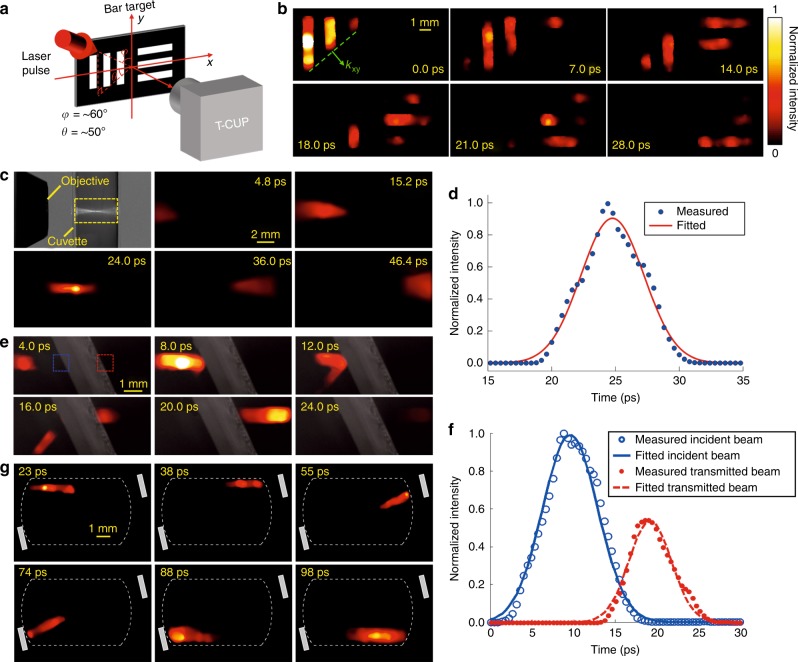
T-CUP for laser pulse sweeping, spatial focusing, reflection, and splitting. **a** Experimental setup for laser pulse sweeping through a scattering bar-pattern target. **b** Representative frames showing a single laser pulse obliquely impinging upon the bar pattern, imaged by T-CUP at 10 Tfps (see Supplementary Movie S3 for the full evolution). In the top left panel, the dashed line indicates the light pulse front, and the arrow denotes the in-plane light propagation direction (*k*_*xy*_). **c** Top left panel: Experimental setup for the spatial focusing of a single laser pulse (532 nm wavelength, 7 ps pulse width) in a weakly scattering aqueous suspension. The 10× objective has a 0.3 NA and a 15 mm focal length. The field of view is indicated by the yellow dashed box. Remaining panels: Representative frames of T-CUP for spatial focusing of a single laser pulse, imaged by T-CUP at 2.5 Tfps (see Supplementary Movie S4 for the full evolution). **d** Time-lapse normalized intensity of the focus with a Gaussian fit. **e** Representative frames showing a single laser pulse (532 nm wavelength, 7 ps pulse duration) split by a 50:50 beam splitter, imaged by T-CUP at 2.5 Tfps (see Supplementary Movie S5 for the full evolution). A small amount of water vapor was sprayed into the path in the air to scatter the light from the scene into the T-CUP system. **f** Time courses of the average normalized intensities on both sides of the beam splitter (the blue and red dashed boxes in the top left panel of **e**). Both time courses were fitted by a Gaussian profile. **g** Representative frames showing a single laser pulse being bounced by two mirrors, imaged by T-CUP at 1 Tfps (see Supplementary Movie S6 for the full evolution). The field of view for T-CUP is indicated by the white dashed box

Four fundamental optical phenomena, namely, a beam sweeping across a surface, spatial focusing, splitting, and reflection, were imaged by the T-CUP system in real time (Fig. 4). In the beam sweeping experiment, a collimated near-infrared ultrashort laser pulse (800 nm wavelength, 50 fs pulse duration) obliquely impinged on a scattering bar pattern. The T-CUP system was placed perpendicular to the target to collect the scattered photons (Fig. 4a). Imaging at 10 Tfps, the T-CUP system clearly reveals how the pulse front of the ultrashort laser pulse swept across the bar pattern (Fig. 4b and Supplementary Movie S3).

In addition, T-CUP enables real-time video recording of spatial focusing of a single picosecond pulse. This phenomenon has been previously documented by phase-contrast microscopy^29^ and interferometry^30^ using conventional pump–probe schemes. In contrast, here, T-CUP was used to capture the scattered light intensity in a single measurement. In the setup, a single laser pulse (532 nm wavelength, 7 ps pulse width) was focused by a 10× objective lens into a weakly scattering aqueous suspension. T-CUP imaged this phenomenon at 2.5 Tfps (Fig. 4c and Supplementary Movie S4). We analyzed the time course of the light intensity at the spatial focus. After normalization, the intensity profile (Fig. 4d) was fitted by a Gaussian function,$\hat I\left( t \right) = {\rm{exp}}\left[ { - 2\left( {t - t_0} \right)^2/\tau _{\rm{g}}^2} \right]$, where *t*_0_ = 24.76 ps, and *τ*_g_ = 4.94 ps. The fitted result yields a 1/*e* width of 6.99 ps, closely matching the experimental specifications.

Imaging at 2.5 Tfps, T-CUP also revealed the spatiotemporal details of the beam splitting process of a single laser pulse (Fig. 4e and Supplementary Movie S5). Impinging on a beam splitter, part of the laser pulse was reflected immediately, while the transmitted portion propagated into the beam splitter and appeared on the other side of the beam splitter after a finite time. To quantitatively analyze the time course of the incident and transmitted pulses, we calculated the average light intensities in the two dashed boxes on both sides of the beam splitter (Fig. 4f). The measured temporal separation between the incident and transmitted pulses was 9.6 ps. Given the 2-mm thickness of this float glass beam splitter (refractive index *n*=1.52 at 532 nm) and the incident angle of ~25°, in theory, the light pulse needs approximately 10 ps to pass through the beam splitter. Thus our measured result agrees well with the theoretical value. It is also noteworthy that the time latency for the reflected and transmitted pulse (9.6 ps) is beyond the imaging capability of previous techniques^23^. T-CUP’s unprecedented frame rate reveals for the first time the spatiotemporal details of this transient event.

Finally, imaging at 1 Tfps, T-CUP was used to capture the reflection of a laser pulse by two mirrors over a sufficiently long time window (Supplementary Movie S6). In Fig. 4g, the first frame shows that the laser pulse has just entered the field of view (FOV). Subsequent frames show the propagating pulse being reflected by the two mirrors before finally traveling out of the FOV. It is noted that an inhomogeneous distribution of scatterers in the aqueous suspension led to increased scattered light intensity in the frames after 74 ps. For this reason, the pulse visually appears to be larger. However, the pulse width, when quantitatively measured via the cross-sectional full width at half maximum, was comparable to that in the rest of the frames.

## Discussion

### Current limitations

The performance of the streak camera, and not the principle of the technique, hinders further increases in frame rate, as well as other important characteristics, such as the spatial resolution and spectral range. The limited performance of the streak camera also impacts the choice of a single-sheared view in the system design (detailed in Supplementary Note 6). Finally, the imaging duty cycle for the T-CUP is currently limited to 5 × 10^–9^–10^–7^ due to the modest sweep frequency (100 fps) and the size of the internal sensor of the streak camera. A precise synchronization is therefore necessary to capture transient events within the time window. A new streak tube design and customized optical components would enable future implementations of a lossless-encoding scheme^24^, which is anticipated to improve the spatial and temporal resolutions in reconstructed images. In addition, the implementations of dual sweep-electrode pairs^31^ and an ultra-large-format camera^32^ are expected to largely increase the duty cycle with the possibility of even realizing continuous streaming.

### Application potential

Single-shot real-time imaging of temporal focusing is expected to immediately benefit the study of nonlinear light–matter interactions. For example, in femtosecond laser 3D micromachining using transparent media (e.g., glass), it was found that temporal focusing can induce an anisotropic fabrication quality^33^ depending on the translation direction of the sample. Thus far, the underlying mechanism for this nonreciprocal writing effect remains elusive. Recent theoretical investigations have indicated a close relation to the plasma dynamics controlled by the tilted pulse front of the temporal focusing pulses^34^. The T-CUP system can substitute for the low-speed cameras that are currently employed in imaging the laser–glass interaction^35^. Specifically, by changing the current zoom imaging system to a 20×, high numerical aperture (NA) objective lens, the microscopic T-CUP system will provide a 10-Tfps frame rate, a 1-µm spatial resolution, and 150-µm FOV at the sample, which is sufficient to simultaneously capture the evolution of a temporally focused pulse and the induced plasma (using a 10×, 0.2-NA objective lens as the focusing lens in Fig. 3a)^10^. The measured spatiotemporal profiles will be analyzed using the established models^36^ to investigate how the pulse front tilt and laser pulse energy affect the transient structure, dispersion properties, and spatial density of the induced plasma. The advantages of single-shot and ultrafast imaging will also pave the way for studying the plasma dynamics generated at microscopically heterogeneous locations (e.g., impurities and defects) in these materials.

Single-shot real-time imaging of temporal focusing by T-CUP also opens up new routes for spatiotemporal characterization of optical waveforms. Currently, temporal microscopes are often deployed as ultrafast all-optical oscilloscopes^2^ to passively analyze optical waveforms with few picosecond temporal resolution^37^ at a specific spatial point. The resolution quantification and imaging experiments in our work have demonstrated that T-CUP, while achieving a comparable temporal resolution, outperforms these oscilloscopes by adding a passive two-spatial-dimensional imaging ability. Thus the large parallel characterization of T-CUP could enable simultaneous ultrafast optical signal processing at multiple wavelengths for telecommunication^38^.

In metrology, a spatiotemporal microscope developed from T-CUP could be well suited for characterizing spatiotemporally complex ultrashort pulses^39^. In many time-resolved high-field laser experiments, the laser systems employed usually have low repetition rates. Therefore, single-shot characterization powered by T-CUP is attractive especially for fast and precise alignment of the setup^40^ and for imaging samples that are difficult to be repeatedly delivered^41^.

In biomedicine, T-CUP holds promise for in vivo tissue imaging. Living biological tissue is an example of dynamic scattering media with a millisecond-level speckle decorrelation time^42^. Thus far, owing to the limited speed of wavefront characterization in existing methods, spatiotemporal focusing beyond the optical diffusion limit has only been realized with static scattering media^43,44^. In contrast, T-CUP demonstrates single-shot femtosecond imaging of transient light patterns in a dynamic scattering medium (Fig. 3c). By integrating T-CUP with interferometry, it is possible to examine the scattered electric field of a broadband beam, which would assist in the design of phase conjugation of spatiotemporal focusing in living biological tissue. Therefore, our work, as an important step in imaging instrumentation, will open up new routes toward deep-tissue wide-field two-photon microscopy, photodynamic therapy, and optogenetics.

### Summary

By improving the frame rate by two orders of magnitude compared with the previous state-of-the-art^23^, T-CUP demonstrated that the ever-lasting pursuit of a higher frame rate is far from ending. As the only detection solution thus far available for passively probing dynamic self-luminescent events at femtosecond timescales in real time, T-CUP was used to reveal spatiotemporal details of transient scattering events that were inaccessible using previous systems. The compressed-sensing-augmented projection extended the application of the Radon transformation to probing spatiotemporal datacubes. This general scheme can be potentially implemented in other imaging modalities, such as tomographic phase microscopy^45^ and time-of-flight volumography^46^. T-CUP’s unprecedented ability for real-time, wide-field, femtosecond-level imaging from the visible to the near-infrared will pave the way for future microscopic investigations of time-dependent optical and electronic properties of novel materials under transient out-of-equilibrium conditions^47^. With continuous improvement in streak camera technologies^48^, future development may enable a 1 quadrillion fps (10^15^ fps) frame rate with a wider imaging spectral range, allowing direct visualization and exploration of irreversible chemical reactions^49^ and nanostructure dynamics^50^.

## Materials and methods

### Summary of the principle of operation of T-CUP

We first derive the expression for the data acquisition of T-CUP in a continuous model. For data acquisition, T-CUP records the intensity distribution of the dynamic scene, *I*(*x, y, t*), in two projected views (Supplementary Fig. S1 and detailed in Supplementary Note 1). The first view, termed the time-unsheared view, directly records the dynamic scene with an external CCD camera (Fig. 2). This recording process is expressed as

4 $$E_{\rm{u}} = {\boldsymbol{TF}}_{\mathbf{u}}I\left( {x,y,t} \right)$$

where *E*_u_ denotes the measured optical energy distribution on the external CCD camera, the linear operator ***F***_**u**_ represents the spatial low-pass filtering in the time-unsheared view, and ***T*** represents the spatiotemporal integration.

The second view, termed the time-sheared view, records the projected view of the spatiotemporal scene from an oblique angle (Supplementary Fig. S1). Specifically, the dynamic scene is first spatially encoded by a pseudo-random binary mask, followed by femtosecond shearing along one spatial axis by a time-varying voltage applied to a pair of sweep electrodes before the scene is finally spatiotemporally integrated on an internal CCD camera in the streak camera. Mathematically, the optical energy measured by the internal CCD camera, *E*_s_, is related to *I*(*x, y, t*) by

5 $$E_{\rm{s}} = {\boldsymbol{TS}}_{\mathbf{f}}{\boldsymbol{DF}}_{\mathbf{s}}{\boldsymbol{C}}I\left( {x,y,t} \right)$$

where the linear operator ***C*** represents spatial encoding, ***F***_s_ represents spatial low-pass filtering in the time-sheared view, ***D*** represents image distortion in the time-sheared view with respect to the time-unsheared view, and ***S***_f_ represents femtosecond shearing.

With the two-view projection, the data acquisition of T-CUP can be described as

6 $$E = {\boldsymbol{O}}I$$

where *E*=[*E*_u_,*αE*_s_]^T^ and ***O***=[***TF***_u_,*α****TS***_f_***DF***_s_***C***]^T^ are the measurement and the linear operators in their concatenated forms, respectively. The scalar factor *α* is the energy calibration ratio between the external CCD camera and the streak camera.

For image reconstruction, we discretized Eqs. 4–6 to obtain Eqs. 1–3 (detailed in Supplementary Note 1). Given the known measurement matrix and leveraging the intrinsic sparsity in the dynamic scene, we estimate that the datacube for the transient scene by solving the inverse problem of Eq. 3. In practice, a two-view reconstruction method, aided by the two-step iterative shrinkage/thresholding algorithm, is implemented to recover the image (detailed in Supplementary Note 2). The T-CUP system greatly improved the reconstruction quality compared with a previously reported CUP system^23^ (illustrated in Supplementary Fig. S2 and detailed in Supplementary Note 3).

### System configuration

The T-CUP system configuration is shown in Fig. 2. The dynamic scene is first imaged by a zoom imaging system built in-house, which supports tunable demagnification ratios of 2–5×. Following the intermediate image, a 50:50 beam splitter sends the incident light in two directions. The reflected beam is recorded by an external CCD camera (Point Grey, GS3-U3-28S4M-C). The transmitted beam is passed onto a DMD (Texas Instruments, LightCrafter 3000) by a 4*f* imaging system with a unit magnification ratio. A pseudo-random binary pattern is displayed on the DMD to encode the input image. As a binary-amplitude spatial light modulator, the DMD consists of hundreds of thousands of micromirrors; each mirror can be tilted to either +12° (as “on” pixels) or –12° (as “off” pixels). The light reflected by the “on” pixels is re-collected by the same 4*f* imaging system. After being reflected by the beam splitter, the spatially encoded dynamic scene is projected onto the entrance port of a femtosecond streak camera (Hamamatsu, C6138). To enable time-resolved measurement in two spatial dimensions, the entrance port is opened to its full width (3 mm). Inside the streak camera, the spatially encoded dynamic scene is first relayed to a photocathode that generates a number of photoelectrons proportional to the light intensity distribution. To temporally shear the spatially encoded dynamic scene, a sweep voltage deflects the photoelectrons to different vertical positions according to their time of flight. The deflected photoelectrons are multiplied by a micro-channel plate and then converted back into light by a phosphor screen. Relayed by output optics, the temporally sheared, spatially encoded dynamic scene is captured by an internal CCD camera (Hamamatsu, ORCA-R2) with 2 × 2 binning (672 × 512 binned pixels, 12.9 × 12.9 μm^2^ binned pixel size). With two-view recording, the light throughput for the T-CUP system is 62.5%.

## Electronic supplementary material

Supplementary Information

Supplementary Movie S1

Supplementary Movie S2

Supplementary Movie S3

Supplementary Movie S4

Supplementary Movie S5

Supplementary Movie S6
