# Supplementary material for: Single-shot real-time femtosecond imaging of temporal focusing
Source: Light Sci Appl. 2018 Aug 8;7:42. doi: 10.1038/s41377-018-0044-7 (PMC6107054; doi:10.1038/s41377-018-0044-7)
Supplement: Supplementary file 1 — Supplementary Information [file 41377_2018_44_MOESM1_ESM.pdf]

**Supplementary Information**

# Single-shot Real-time Femtosecond Imaging of Temporal Focusing

Jinyang Liang[1,2,†], Liren Zhu[1,†] and Lihong V. Wang[1,*]

[1]Caltech Optical Imaging Laboratory, Andrew and Peggy Cherng Department of Medical Engineering, Department of Electrical Engineering, California Institute of Technology, 1200 East California Boulevard, Mail Code 138-78, Pasadena, CA 91125, USA

[2]Present address: Centre Énergie Matériaux Télécommunications, Institut National de la Recherche Scientifique, 1650 boulevard Lionel-Boulet, Varennes, QC J3X1S2, Canada

*Corresponding author: LVW@caltech.edu

[†]These authors contributed equally to this work.

## Supplementary Notes

*Supplementary Notes 1: Derivation of T-CUP's data acquisition*

T-CUP's data acquisition records a time-unsheared view and a time-sheared view of the dynamic scene (Fig. S1). For simplicity of notation, we here make the following assumptions. First, the imaging system has a unit magnification. Second, the dynamic scene can be perfectly imaged to the DMD. Third, the external CCD camera, the internal CCD camera of the streak camera, and the DMD have matched pixels and the same pixel dimension, denoted as $d$. Fourth, we only explicitly express the time delay due to the streak camera.

In the following, we first derive the expression of T-CUP's data acquisition in the continuous model and then discretize it for the image reconstruction algorithm. In the time-unsheared view, the intensity distribution of the dynamic scene on the external CCD camera can be expressed as

$$I_{F_{\mathrm{u}}}(x'_{\mathrm{u}}, y'_{\mathrm{u}}, t) = \boldsymbol{F}_{\mathbf{u}}\{I(x, y, t)\}\,, \tag{S1}$$

where the subscript "u" stands for "unsheared", and $\boldsymbol{F}_{\mathbf{u}}$ denotes the spatial low-pass filtering caused primarily by the external CCD camera's lens. With the spatiotemporal integration operator $\boldsymbol{T}$, the optical energy measured by the $[m, n]$ pixel on the CCD camera is given by

$$E_{\mathrm{u}}[m,n] = \boldsymbol{T}\{I_{F_{\mathrm{u}}}(x'_{\mathrm{u}}, y'_{\mathrm{u}}, t)\}$$
$$= \int dx'_{\mathrm{u}} \int dy'_{\mathrm{u}} \left\{\left[\int dt\, I_{F_{\mathrm{u}}}(x'_{\mathrm{u}}, y'_{\mathrm{u}}, t)\right] \cdot \mathrm{rect}\left[\frac{x'_{\mathrm{u}}}{d} - \left(m + \frac{1}{2}\right), \frac{y'_{\mathrm{u}}}{d} - \left(n + \frac{1}{2}\right)\right]\right\}. \quad \text{(S2)}$$

In the time-sheared view, $I(x, y, t)$ is first spatially encoded by a pseudo-random binary pattern $c(x, y)$, which can be expressed as

$$I_C(x, y, t) = c(x, y) I(x, y, t)\,. \quad \text{(S3)}$$

Here, $I_C(x, y, t)$ represents the intensity distribution of the spatially encoded dynamic scene, which is then relayed to the entrance port of a streak camera and further imaged to the photocathode by the input optics inside the streak camera. This process can be expressed as

$$I_{F_{\mathrm{s}}}(x, y, t) = \boldsymbol{F}_{\mathbf{s}}\{I_C(x, y, t)\}\,, \quad \text{(S4)}$$

where $\boldsymbol{F}_{\mathbf{s}}$ denotes the spatial low-pass filtering due to the input optics in the streak camera. Then, an image distortion operator is applied to $I_{F_{\mathrm{s}}}(x, y, t)$:

$$I_{\mathrm{D}}(x, y, t) = \boldsymbol{D}\{I_{F_{\mathrm{s}}}(x, y, t)\}\,, \quad \text{(S5)}$$

where $\boldsymbol{D}$ accounts for image distortion in the time-sheared view with respect to the time-unsheared view.

Next, the dynamic scene enters the streak tube [1]. The operation of the streak tube introduces a time delay, $\tau_{\mathrm{sc}}$, and we define the new time axis $t' = t + \tau_{\mathrm{sc}}$. In addition, we define the new spatial axes $x' = x$ and $y' = y - vt$, where $v$ denotes the shearing velocity of the streak camera. Under the new coordinate system, the temporal shearing along the vertical spatial axis is given by

$$I_{\mathrm{S}}(x', y', t') = \boldsymbol{S}_{\mathbf{f}}\{I_{\mathrm{D}}(x, y, t)\} = I_{\mathrm{D}}(x', y' + v(t' - \tau_{\mathrm{sc}}), t' - \tau_{\mathrm{sc}})\,. \quad \text{(S6)}$$

Finally, $I_\mathrm{S}(x', y', t')$ is imaged to an internal CCD camera inside the streak camera. The optical energy measured by the pixel $[m, n]$ on the CCD camera takes the form of

$$E_\mathrm{s}[m,n] = \boldsymbol{T}\{I_\mathrm{S}(x',y',t')\}$$
$$= \int dx' \int dy' \left\{ \left[ \int dt'\, I_\mathrm{S}(x',y',t') \right] \cdot \mathrm{rect}\left[ \frac{x'}{d} - \left(m + \frac{1}{2}\right), \frac{y'}{d} - \left(n + \frac{1}{2}\right) \right] \right\}. \quad \text{(S7)}$$

To recover the dynamic scene using a compressed-sensing-based reconstruction algorithm, we derive the discrete form of T-CUP's data acquisition. To simplify the notation, we start by discretizing the dynamic scene with a voxel size of $(d, d, \tau_\mathrm{s})$, where $\tau_\mathrm{s} = d/v$. The discrete dynamic scene can be expressed by

$$I[m,n,k] = \int dt \int dx \int dy\, I(x,y,t) \quad \text{(S8)}$$
$$\cdot\, \mathrm{rect}\left[ \frac{x}{d} - \left(m + \frac{1}{2}\right), \frac{y}{d} - \left(n + \frac{1}{2}\right), \frac{t}{\tau_s} - \left(k + \frac{1}{2}\right) \right].$$

The measured optical energy distribution in the time-unsheared view (Eq. S2) can be approximated by the following discrete form:

$$E_\mathrm{u}[m,n] = \frac{d^3}{v} \sum_k (h_\mathrm{u} * I)[m,n,k]\,, \quad \text{(S9)}$$

where $h_\mathrm{u}$ is the discrete convolution kernel of the operator $\boldsymbol{F}_\mathrm{u}$, and $*$ denotes the discrete two-dimensional (2D) spatial convolution operation. Equation S9 represents a single-angle Radon transform of the dynamic scene, with the direction of projection parallel with the time axis.

For the time-sheared view, the encoding mask is discretized by

$$c[m,n] = \int dx \int dy\, c(x,y) \cdot \mathrm{rect}\left[ \frac{x}{d} - \left(m + \frac{1}{2}\right), \frac{y}{d} - \left(n + \frac{1}{2}\right) \right], \quad \text{(S10)}$$

and the spatially encoded dynamic scene can be expressed by

$$I_\mathrm{C}[m,n,k] = c[m,n] \cdot I[m,n,k]\,. \quad \text{(S11)}$$

Thus, the measured optical energy distribution in the time-sheared view (Eq. S7) can be discretized by

$$E_{\mathrm{s}}[m,n] = \frac{d^3}{v}\sum_{k}(h_{\mathrm{s}} * I_{\mathrm{C}})[f_{\mathrm{D}}, g_{\mathrm{D}} + k, k]\,, \tag{S12}$$

where $h_{\mathrm{s}}$ is the discrete convolution kernel of the operator $\boldsymbol{F}_{\mathrm{s}}$, while $f_{\mathrm{D}}$ and $g_{\mathrm{D}}$ are the discrete coordinates transformed from $m$ and $n$, according to the operator $\boldsymbol{D}$, determined by a calibrated 2D projective transformation [2]. Equation S12 represents a Radon transform from an oblique angle determined by $\tan^{-1}[d/(\tau_{\mathrm{s}}v)]$.

*Supplementary Note 2: Details of T-CUP's image reconstruction*

Provided with prior knowledge of the linear operator $\boldsymbol{O}$ in the data acquisition process, we can recover the datacube of the input scene from the two-view measurements by solving the inverse problem of Eq. M3. Given the sparsity of the input scene, compressed-sensing algorithms can be implemented to solve the following minimization problem:

$$\hat{I} = \arg\min_{I}\left[\frac{1}{2}\|E - \boldsymbol{O}I\|_2^2 + \rho\Phi(I)\right], \tag{S13}$$

where $E = [E_{\mathrm{u}}, \alpha E_{\mathrm{s}}]^T$ is the measurement in the concatenated form. $\alpha$ is a scalar factor introduced to balance the energy ratio between the two views during measurement. $\Phi(I)$ is a regularization function promoting sparsity, and the regularization parameter $\rho$ adjusts the weight between fidelity and sparsity. In T-CUP's image reconstruction, we employed a two-view reconstruction algorithm, which was aided by the two-step iterative shrinkage/thresholding (TwIST) algorithm [3]. The three-dimensional total variation was used as the regularization function [4].

The implementation of the two-view reconstruction algorithm requires both the overall system operator $\boldsymbol{O}$ and its adjoint operator $\boldsymbol{O}^* = [\boldsymbol{F}_{\mathbf{u}}^* \boldsymbol{T}^*, \alpha \boldsymbol{C}^* \boldsymbol{F}_{\mathbf{s}}^* \boldsymbol{D}^* (\boldsymbol{T}\boldsymbol{S}_{\mathbf{f}})^*]^T$. The detailed procedure of calculating the adjoint of each operator in $\boldsymbol{O}$ has been discussed elsewhere [2,4].

*Supplementary Note 3: Simulation test of the two-view reconstruction algorithm*

To test the two-view reconstruction algorithm, we numerically simulated a dynamic scene, shown in Fig. S2a. Specifically, this dynamic scene contained 10 frames, each with a size of 200 × 200 pixels. A Shepp-Logan (S-L) phantom moved from left to right at four pixels per frame and flashed at the third, fifth, and eighth frames. The other frames were left black and filled with zero intensity. The time-unsheared view and the time-sheared view were generated according to T-CUP's data acquisition model. Shot noise was added to the final 2D images to emulate measurement.

To demonstrate the advantages of our method, we reconstructed the dynamic scene using two approaches: conventional compressed-sensing-based single-view reconstruction [4] and the new compressed-sensing-augmented two-view reconstruction. The simulated reconstruction results are shown in Fig. S2b–c. As a quantitative comparison, we computed the root-mean-square error (RMSE) of each frame. RMSE is defined as

$$\sigma_k = \sqrt{\frac{1}{N_{\mathrm{p}}} \sum (I_{\mathrm{r}}[m,n,k] - I[m,n,k])^2}\,, \qquad \text{(S14)}$$

where $I_{\mathrm{r}}$ is the reconstructed dynamic scene, and $N_{\mathrm{p}}$ is the number of pixels in each frame. We compared the image reconstruction errors of both methods and plotted the RMSE of each individual frame (Fig. S2d). The new reconstruction algorithm achieved a considerably smaller reconstruction error in all frames. Specifically, the residual energy in the supposedly black frames

was greatly reduced, improving the contrast along the time axis, even though the additional time-unsheared view did not directly provide temporal resolution.

*Supplementary Note 4: Characterization of T-CUP's spatial and temporal resolutions*

To quantify T-CUP system's spatial and temporal resolutions, we imaged a dynamic scene: a single 50-fs laser pulse obliquely illuminated a scattering spoke pattern (Fig. S3a). Positioned perpendicularly to the pattern's surface, the T-CUP system imaged this dynamic scene using four frame rates: 0.5, 1, 2.5, and 10 Tfps. Each reconstructed $(x, y, t)$ datacube was temporally projected onto the $x$-$y$ plane. The projected image was then Fourier transformed to calculate the spatial frequency response (Fig. S3b). We defined the spatial resolution as the noise-limited bandwidth at the $2\sigma$ threshold above the average background, where the noise $\sigma$ was defined as the standard deviation of the background. Spatial resolutions at all four angular branches (i.e., 0°, 30°, 60°, and 90°) were computed for each frame rate. To compare these results quantitatively, we calculated the degradation ratio between the spatial resolution in the temporally projected image at each frame rate and that in the reference image (Fig. S3c). T-CUP's spatial resolution at 10 Tfps is decreased by a factor of 1.25, compared with its spatial resolution at 0.5 Tfps. In addition, for each frame rate, a small resolution anisotropy among different angular branches is observed, which is attributed to the spatiotemporal mixing only along the $y$-axis.

The temporal resolution was quantified by averaging the full widths at half maximum of the temporal responses of one randomly selected pixel in the spoke image. To perform a quantitative comparison, we also calculated the degradation ratio by multiplying the temporal resolution with the corresponding frame rate (Fig. S3d). At 0.5 Tfps, the degradation ratio is 3.17. This degradation

is attributed to the finite pixel size in the spatial encoding mask [5]. The degradation ratio increases at higher frame rates, reaching 5.75 at 10 Tfps.

The larger degradation ratios of T-CUP's spatial and temporal resolutions at higher frame rates were imputed to the reduced performance of the streak camera. First, to maintain a sufficient signal-to-noise ratio, higher gains in the micro-channel plate were used for higher frame rates, which led to a larger amplification noise. Second, the trajectory length difference of photoelectrons induced image distortion. Photoelectrons that came from the edges of the photocathode had a longer path to travel than the ones coming from the center. The resultant time-of-arrival difference misplaces the photoelectrons to undesired vertical positions, deteriorating the ensuing image reconstruction. In addition, higher frame rates resulted in a larger deflection along the *y*-axis, which also increased the photoelectrons' path differences and thus aggravated defocusing.

Even if the streak camera could form a perfect image without any geometric aberration and electronic noise, the spatial and temporal resolutions of the T-CUP camera would, nevertheless, still be limited by two other factors. First, there is a spread in the initial velocity of photoelectrons during photon-to-electron conversion, which is imposed by the photocathode material. This velocity spread would transfer to variation in photoelectrons' times of arrival, which significantly impacts the temporal resolution. Second, the T-CUP camera—leveraging on the single-shot 2D imaging of the streak camera—produced far more photoelectrons at a given time point than those generated from the conventional use of the streak camera in multi-shot one-dimensional streak imaging. The larger number of photoelectrons could lead to a stronger space-charge effect, which would blur the photoelectronic image and also reduce the temporal resolution.

In summary, we have characterized T-CUP's spatial and temporal resolutions at multiple frame rates in a macroscopic imaging setup. Although not demonstrated here, the T-CUP system's spatial

resolution and the field of view (FOV) can be easily scaled by replacing the zoom imaging system (Fig. 2) with other optical components, such as a microscope objective lens. In this regard, microscopic or mesoscopic T-CUP systems can be easily designed for future investigations.

*Supplementary Note 5: Theory and simulation of temporal focusing*

Here we derive the equation for temporal focusing. The coordinates are marked in Fig. 3a in the main text. The analysis is restricted in the $y$- $\tau$ plane at various positions on the $z$-axis, where $\tau = t - z/c$ is the time delay. In addition, the $x$-dependence is ignored [6]. The incident Gaussian pulse is modeled as

$$\mathrm{E_i}(y,\tau) = \exp\left\{-\left[\left(\frac{y}{y_\mathrm{i}}\right)^2 + \left(\frac{\tau}{\tau_\mathrm{i}}\right)^2\right]\right\}, \tag{S15}$$

where $y_\mathrm{i}$ is the spatial width on the $y$-axis, and $\tau_\mathrm{i}$ is the pulse duration. The temporal frequency spectrum of the incident Gaussian pulse, $\mathrm{E_i}(y,\omega)$, is given by

$$\mathrm{E_i}(y,\omega) = \int_{-\infty}^{\infty} \mathrm{E_i}(y,\tau)\exp(-i\omega\tau)d\tau\,, \tag{S16}$$

where $\omega$ denotes the temporal frequency. The incident Gaussian pulse illuminates a ruled grating with a groove spacing $d_\mathrm{g}$, which induces a linear phase [7]

$$\Psi_\mathrm{g}(y,\omega) = \exp(ik\beta\omega y)\,, \tag{S17}$$

where $k_\mathrm{o} = 2\pi/\lambda$ is the wavenumber, and $\lambda$ is the wavelength. $\beta = -\frac{m\lambda^2}{2\pi c d_\mathrm{g}\cos\theta}$ is the angular dispersion parameter of the grating, where $m$ is the diffraction order, $c$ the speed of light, and $\theta$ the diffraction angle. Right after the grating, the pulse profile is given by

$$\mathrm{E_g}(y,\tau) = \exp\left\{-\left[\left(\frac{y}{y_\mathrm{i}}\right)^2 + \left(\frac{\tau - k_\mathrm{o}\beta y}{\tau_\mathrm{i}}\right)^2\right]\right\}. \tag{S18}$$

The $y$-$\tau$ coupling in Eq. S18 shows the pulse front tilt induced by the angular dispersion. The tilt angle after the grating is given by $\gamma_{\mathrm{g}} = \tan^{-1}(k_{\mathrm{o}} c\beta)$. After the grating, this pulse propagates through a $4f$ imaging system (Fig. 3a). The focal lengths of the collimating lens and focusing lens are denoted as $f_1$ and $f_2$, respectively. Mathematically, the pulse experiences two Fourier transformations. At the focal plane of the focusing lens, the pulse profile $\mathrm{E_o}(y, \tau)$ can be expressed as

$$\mathrm{E_o}(y, \tau) = \exp\left\{-\left[\left(\frac{y}{My_{\mathrm{i}}}\right)^2 + \left(\frac{\tau + k_{\mathrm{o}}\beta y/M}{\tau_{\mathrm{i}}}\right)^2\right]\right\}, \tag{S19}$$

where $M$ is the magnification ratio. Eq. S19 can be regarded as the image of the pulse profile immediately after the grating (Eq. S18). The imaging system scales the pulse's spatial dimension on the $y$-axis by $M$ times. As a result, the tilt angle on the image plane, $\gamma_{\mathrm{o}}$, is given by $\gamma_{\mathrm{o}} = \tan^{-1}(-k_{\mathrm{o}} c\beta/M)$.

We also numerically simulated temporal focusing using measured experimental parameters. The incident ultrashort laser pulse (Eq. S15) had a pulse width $\tau_{\mathrm{i}} = 50$ fs with a central wavelength $\lambda_c = 800$ nm. Pulse's sizes on the $x$-axis and $y$-axis were set to $x_{\mathrm{i}} = 1$ mm and $y_{\mathrm{i}} = 3$ mm. This femtosecond laser pulse illuminated a 1200 line mm$^{-1}$ grating (groove spacing $d_{\mathrm{g}} = 0.83$ μm). The first-order diffracted beam ($m_{\mathrm{d}} = 1$) was collinear with the $z$-axis ($\theta = 0°$) with a simulated pulse-front tilt $\gamma_{\mathrm{g}} = 43.8°$.

The diffracted beam propagated from the diffraction grating to the collimation lens, which was simulated using the Fresnel diffraction theory [8]. Immediately before the collimation lens, the electric field of the diffracted beam is expressed by

$$\mathrm{E}_{\mathrm{bf_1}}(k_y, \omega) = \mathrm{E_g}(k_y, \omega) \cdot \mathrm{H}(k_y, \omega), \tag{S20}$$

where $\mathrm{E_g}(k_y, \omega)$ is the Fourier transform of $\mathrm{E_g}(y, \tau)$, i.e., $\mathrm{E_g}(k_y, \omega) = \mathcal{F}\{\mathrm{E_g}(y, \tau)\}$, and $\mathrm{H}(k_y, \omega) = \exp\left[-i2cf_1 \left(\pi k_y\right)^2/\omega\right]$ is the propagation phase factor. The focal length of the lens was $f_1 = 200$ mm. For simplicity of notation here, we have dropped the constant phase term in the Fresnel diffraction [9].

The collimation lens added a phase factor into the diffracted beam. The electric field immediately after the lens can be expressed by

$$\mathrm{E_{af_1}}(y, \tau) = \mathrm{E_{bf_1}}(y, \tau) \cdot \mathrm{t_{f_1}}(y)\,, \tag{S21}$$

where $\mathrm{t_{f_1}}(y) = \exp[-i\omega y^2/(2f_1 c)]$ is the phase profile of the lens. After the collimation lens, the diffracted beam continued propagating to the focusing lens (focal length $f_2 = 50$ mm), which focused it on the temporal focusing plane. The beam propagation process was simulated by following the same procedure as above. With a magnification ratio $M = 1/4$, the pulse-front tilt at the temporal focus was $\gamma_\mathrm{o} = 75.4°$.

Finally, we converted the simulation data from the $y$-$z$-$\tau$ coordinates to the $y$-$z$-$t$ coordinates. Figure S4a shows five representative frames of a simulated spatially chirped femtosecond pulse impinging on the temporal focusing plane, observed in front view at 2.5 Tfps and in a 1.75 mm × 1.75 mm $(x, y)$ FOV. These frames show the pulse on the temporal focusing plane reproduces the same temporal width (50 fs) as the incident pulse. However, with a tilted pulse front, this pulse sweeps through the temporal focusing along the $y$-axis. Figure S4b shows five representative frames of the simulated temporal focusing in a 1.75 mm × 4.5 mm $(y, z)$ FOV. To account for the spatial resolution of the observing optics used in our experiments, a 2D, square Hamming window with a full width at half maximum of 0.24 mm was used to blur the simulated side-view and front-view images. The simulated frame rate was 2.5 Tfps. These frames show the shortest pulse width on the temporal focusing plane. In addition, similar to the front-view imaging, the sweeping along

the $y$-axis can be clearly observed. A corresponding movie shows the simulated images of the front side views synchronously frame by frame in Movie S1. Finally, to clearly render the temporal focusing process, spatial transformation was implemented so that the pulse front appeared to be perpendicular to the propagation direction (Fig. S5). Specifically, each frame of the side-view movie was rotated by an angle of $\gamma_{\mathrm{o}}$ clockwise about the intersection of the temporal focusing plane and the $z$ axis, and shifted by $c\Delta t \sin\gamma_{\mathrm{o}}$ ($\Delta t$ is the time interval between frames) upwards in the vertical direction. The corresponding movie is shown as Movie S2.

*Supplementary Note 6: Rationales for T-CUP's imaging system design*

The current T-CUP system was designed by considering the limited performance of the existing devices and the experimental requirements for imaging temporal focusing. First, the streak camera used in this work (Hamamatsu C6138) trades spatial and temporal resolutions as well as dynamic range for higher frame rates and higher incident intensity [10], owing mainly to the design of the streak tube [11]. The 2.5 Tfps frame rate was selected by balancing the spatial and temporal resolutions and the frame rate. At this speed, the streak camera had a spatial resolution of ~10 lp $mm^{-1}$ in conventional operating (i.e., 1D streaking) mode. At the 2.5-Tfps frame rate, light travels 120 µm in each frame. For blur-free observation with satisfied Nyquist sampling, a demagnification ratio of two from the object to the photocathode was thus used.

The intra-frame light traveling distance also determined the numerical aperture of the temporal focusing setup (Fig. 3a) to be ~0.08. Among existing off-the-shelf diffraction gratings, we selected the one with a 1200 lines $mm^{-1}$ density due to design priorities including compact system size, maximized image size, and minimized image aberration. Therefore, considering the ~30 nm bandwidth of the incident femtosecond laser pulse, the magnification ratio of the temporal focusing

setup [Fig. 3(a)] was determined to be $M = 1/4$, which produced a pulse front tilt of ~76°. To capture the entire temporal focusing process in the designed FOV, the incident pulse dimension was thus limited to $y_{\mathrm{i}}$ = 3 mm.

With this design in mind, we forewent the implementation of the lossless-encoding (LLE) scheme [2] on the T-CUP system because it could not provide a sufficient FOV for temporal focusing. Specifically, the input imaging plane of the streak camera, which was several millimeters inside the camera's head, was inaccessible in free space. The right-angle prism mirror (RAPM) thus had to be placed a certain distance away from the imaging plane. Because rays converged to the imaging plane, a small gap (~2 mm) was created between the two views. For the streak camera (Hamamatsu C7700) used in the previous CUP systems, its relatively large photocathode size (17 mm × 5 mm) could tolerate this gap. The larger image size also would help to reduce the gap size. However, for the streak camera (Hamamatsu C6138) used in this work, its small photocathode (with a diameter of 3 mm) presented challenges in the design. In addition, the large size of the camera's head (with a width of ~150 mm) forbade placing optical components to their optimal positions. Thus, our attempt to implement the LLE design with a unity magnification led to an FOV of approximately 0.75 × 0.75 $mm^2$ at the photocathode. Attempts were made by using an imaging system to relay the image formed immediately after RAPM. However, the edge of the RAPM brought in large distortion and vignetting, which equivalently restricted the FOV. The severely limited FOV could not satisfy the aforementioned requirements for imaging temporal focusing. Therefore, we implemented the scheme to detect a single time-sheared view.

System improvement is possible from two aspects. First, a new streak camera with a minimized space-charge effect could partially resolve the spatial and temporal resolutions' degradation at a

higher photon flux. In addition, with precise alignment, customized optics with relay optics should improve the FOV at the photocathode.

## References


1 Kinoshita, K., Ito, M. & Suzuki, Y. Femtosecond streak tube. *Rev. Sci. Instrum.* **58**, 932-938 (1987).

2 Liang, J. *et al.* Single-shot real-time video recording of photonic Mach cone induced by a scattered light pulse. *Sci. Adv.* **3**, e1601814 (2017).

3 Bioucas-Dias, J. M. & Figueiredo, M. A. T. A New TwIST: Two-Step Iterative Shrinkage/Thresholding Algorithms for Image Restoration. *IEEE Trans. Image Process.* **16**, 2992-3004 (2007).

4 Gao, L., Liang, J., Li, C. & Wang, L. V. Single-shot compressed ultrafast photography at one hundred billion frames per second. *Nature* **516**, 74-77 (2014).

5 Zhu, L. *et al.* Space- and intensity-constrained reconstruction for compressed ultrafast photography. *Optica* **3**, 694-697 (2016).

6 Boyd, R. W. *Nonlinear optics*. (Academic press, 2003).

7 Martinez, O. E. Pulse distortions in tilted pulse schemes for ultrashort pulses. *Opt. Commun.* **59**, 229-232 (1986).

8 Liang, J. & Becker, M. F. Spatial bandwidth analysis of fast backward Fresnel diffraction for precise computer-generated hologram design. *Appl. Opt.* **53**, G84-G94 (2014).

9 Goodman, J. W. *Introduction to Fourier optics*. (Roberts and Company Publishers, 2005).

10 Hamamatsu. *FESCA-200 Femtosecond streak camera*, <https://www.hamamatsu.com/resources/pdf/sys/SHSS0003E_C6138.pdf> (2015).

11 Takahashi, A., Nishizawa, M., Inagaki, Y., Koishi, M. & Kinoshita, K.New femtosecond streak camera with temporal resolution of 180 fs *Proc. SPIE* **2116**, 10 (1994).

## Supplementary figures

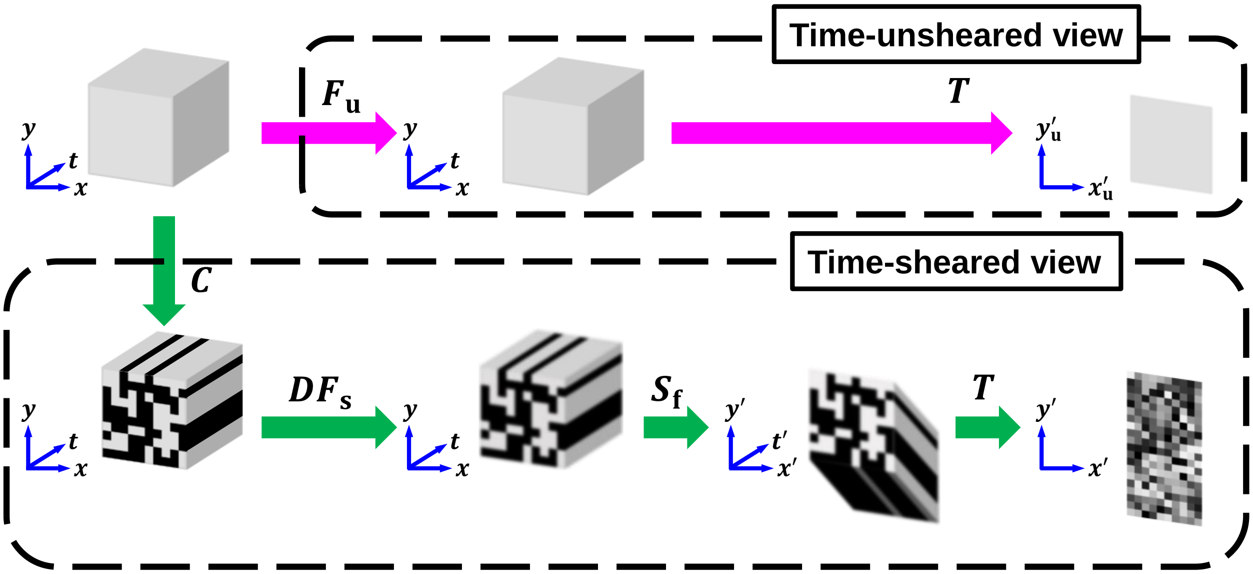


**Figure S1. Schematic of T-CUP's data acquisition.** $x$, $y$, spatial coordinates of the scene; $x'_u$, $y'_u$, spatial coordinates of the external CCD camera; $x'$, $y'$spatial coordinates of the streak camera; $t$, $t'$, time; $\boldsymbol{C}$, spatial encoding operator; $\boldsymbol{F}_u$ and $\boldsymbol{F}_s$, spatial low-pass filtering operators, which blur the images as indicated; $\boldsymbol{D}$, image distortion operator; $\boldsymbol{S}_f$, femtosecond shearing operator; $\boldsymbol{T}$, spatiotemporal integration operator.

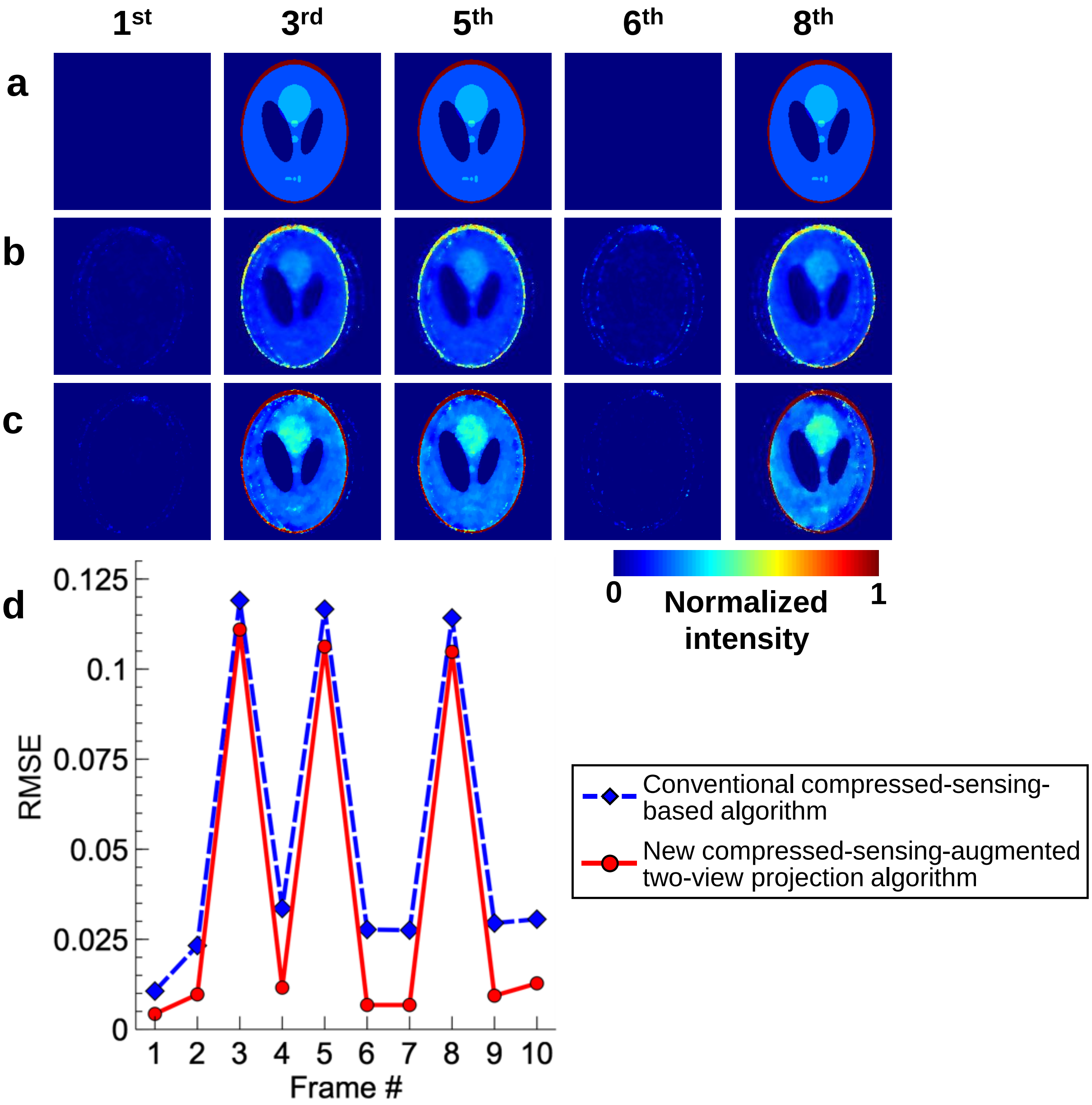


**Figure S2. Results of numerical simulation of the compressed-sensing-augmented two-view reconstruction algorithm.** (**a**) Selected frames from the ground truth dynamic scene. (**b**) Selected frames from the conventional compressed-sensing-based reconstruction movie. (**c**) Selected frames from the compressed-sensing-augmented two-view reconstruction movie. (**d**) Comparison of the root-mean-square errors (RMSE) of frames based on the conventional and new reconstruction algorithms.

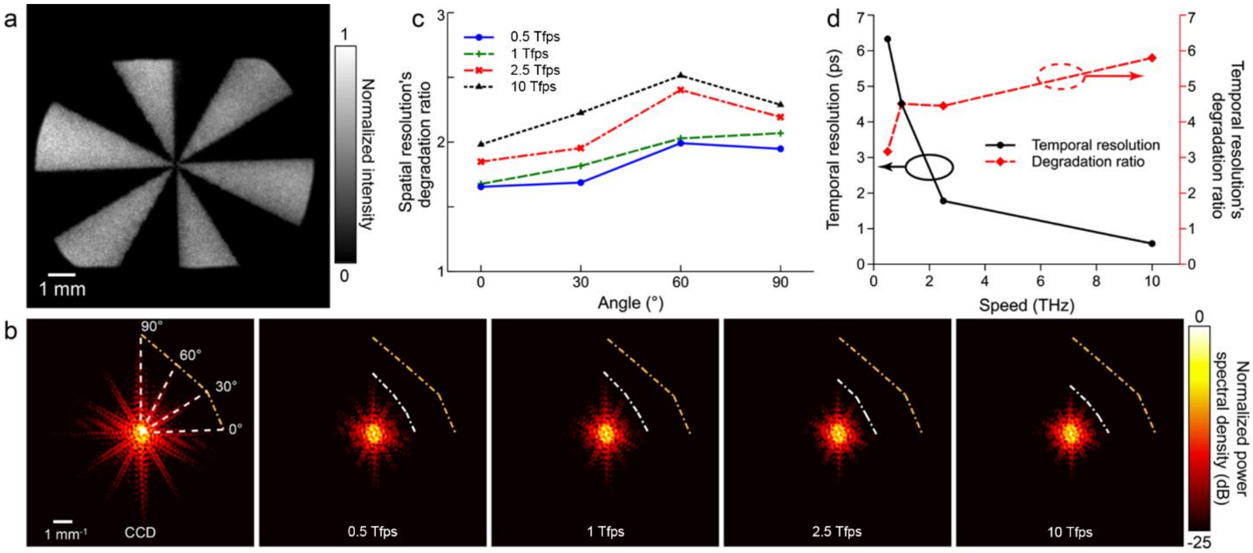


**Figure S3. Characterization of T-CUP's spatial and temporal resolutions at frame rates of 0.5, 1, 2.5, and 10 Tfps. (a)** Reference image of the spoke pattern captured by the external CCD camera. This image was coregistered to the streak camera's view. The circular boundary was imposed by that of the streak camera's photocathode. **(b)** Spatial frequency responses of the reference image and the temporally projected images at the four frame rates. The white and orange dash-dotted lines represent band limits of the T-CUP system and the front optics, respectively. **(c)** Spatial resolution's degradation ratio as a function of angles at the four frame rates. **(d)** Temporal resolution and its degradation ratio at the four frame rates.

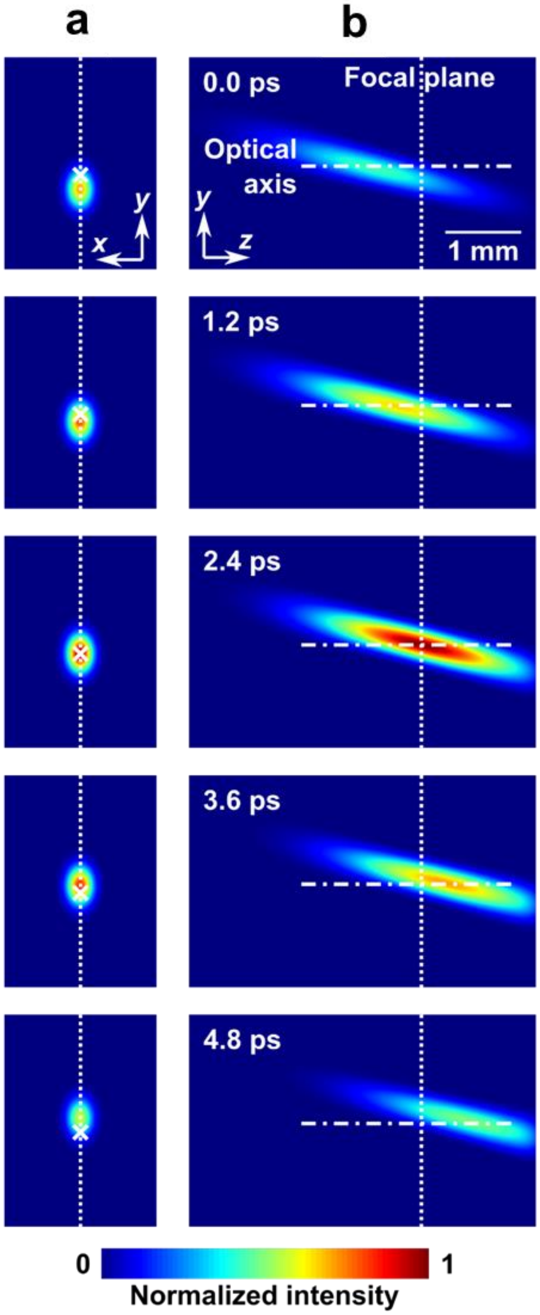


**Figure S4. Simulation of front-view (a) and side-view (b) detections of temporal focusing.** The pulse width was 50 fs; the central frequency was 800 nm; $(x, y)$ beam size on the temporal focusing plane was 0.25 mm and 1.5 mm, respectively. This pulse propagated through the system described in Fig. 3a. The frame rate in the simulation was 2.5 Tfps.

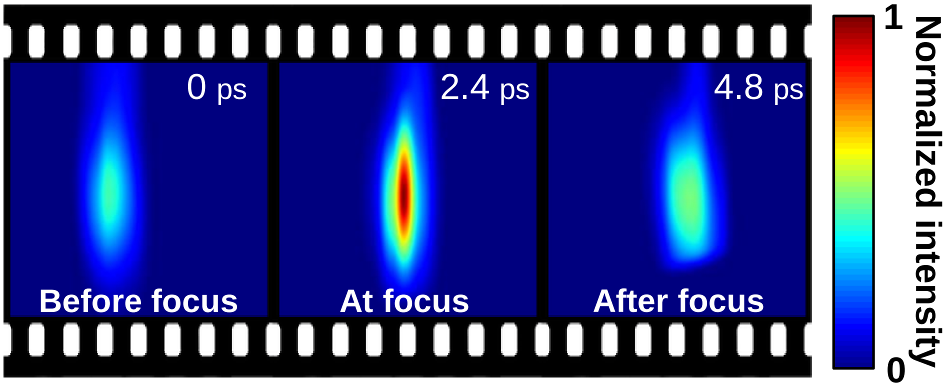


**Figure S5. Representative frames of temporal focusing rendered with spatial transformation.** The corresponding movie is shown as Movie S2.

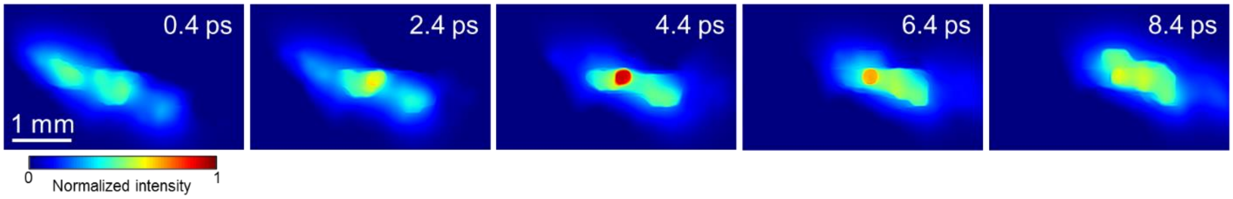


**Figure S6. Another dataset of temporal focusing imaging from the side.**

**Supplementary Movies**

**Movie S1:** T-CUP of temporal focusing in both front and side views at 2.5 Tfps. The simulated and experimental movies are synchronized frame by frame.

**Movie S2:** T-CUP of temporal focusing in side view at 2.5 Tfps, with a spatial transformation to render a vertical pulse front for better presentation. The simulated and experimental movies are synchronized frame by frame.

**Movie S3:** T-CUP of laser pulse sweeping across a bar pattern at 10 Tfps.

**Movie S4:** T-CUP of spatial focusing at 2.5 Tfps.

**Movie S5:** T-CUP of laser pulse splitting at 2.5 Tfps.

**Movie S6:** T-CUP of laser pulse reflection by two mirrors at 1 Tfps.
